# [Corrigendum] Arginine ADP-ribosyltransferase 1 promotes angiogenesis in colorectal cancer via the PI3K/Akt pathway

**DOI:** 10.3892/ijmm.2026.5914

**Published:** 2026-07-02

**Authors:** Lian Yang, Ming Xiao, Xian Li, Yi Tang, Ya-Lan Wang

Int J Mol Med 37: 734-742, 2016; DOI: 10.3892/ijmm.2016.2473

Following the publication of the above paper, it has been drawn to the Editor's attention by a concerned reader that, regarding the cell migration assay data shown in Fig. 3A on p. 738, data panels 'a' and 'c' for the 'Co-cultured for 12 h' experiments (lower row of panels) exhibited overlapping sections, such that these data panels, which were intended to show the results of differently performed experiments, had apparently been derived from the same original source. Moreover, the 'Co-cultured for 6 h'/'d' panel (on the top row) was also found to share overlapping data with the 'Co-cultured for 12 h'/'b' panel on the lower row. In addition, it was noted that various of the β-actin control blots shown in Figs. 1B and 6A on p. 737 and p. 740, respectively, were strikingly similar, where different experimental conditions were reported, suggesting that either or both of these figures may have been assembled incorrectly.

Upon examining their original data, the authors have realized that data in these figures were inadvertently assembled incorrectly. Certain of the images for the 6 h and 12 h time points were inadvertently misused during figure compilation due to a folder selection error; specifically, the data erroneously shown in the 'Co-cultured for 6 h'/'d' panel and in the 'Co-cultured for 12 h'/'a' panel have been replaced with the correct data. Concerning the re-use of the β-actin control blots, those in Fig. 6 were included in error; the revised versions of Figs. 4 and 6 are shown on the next page. The authors confirm that the errors associated with these figures did not have any significant impact on either the results or the conclusions reported in this study, and all the authors agree with the publication of this Corrigendum. The authors are grateful to the Editor of *International Journal of Molecular Medicine* for allowing them the opportunity to publish this Corrigendum; furthermore, they apologize to the readership of the Journal for any inconvenience caused.

## Figures and Tables

**Figure 3:**
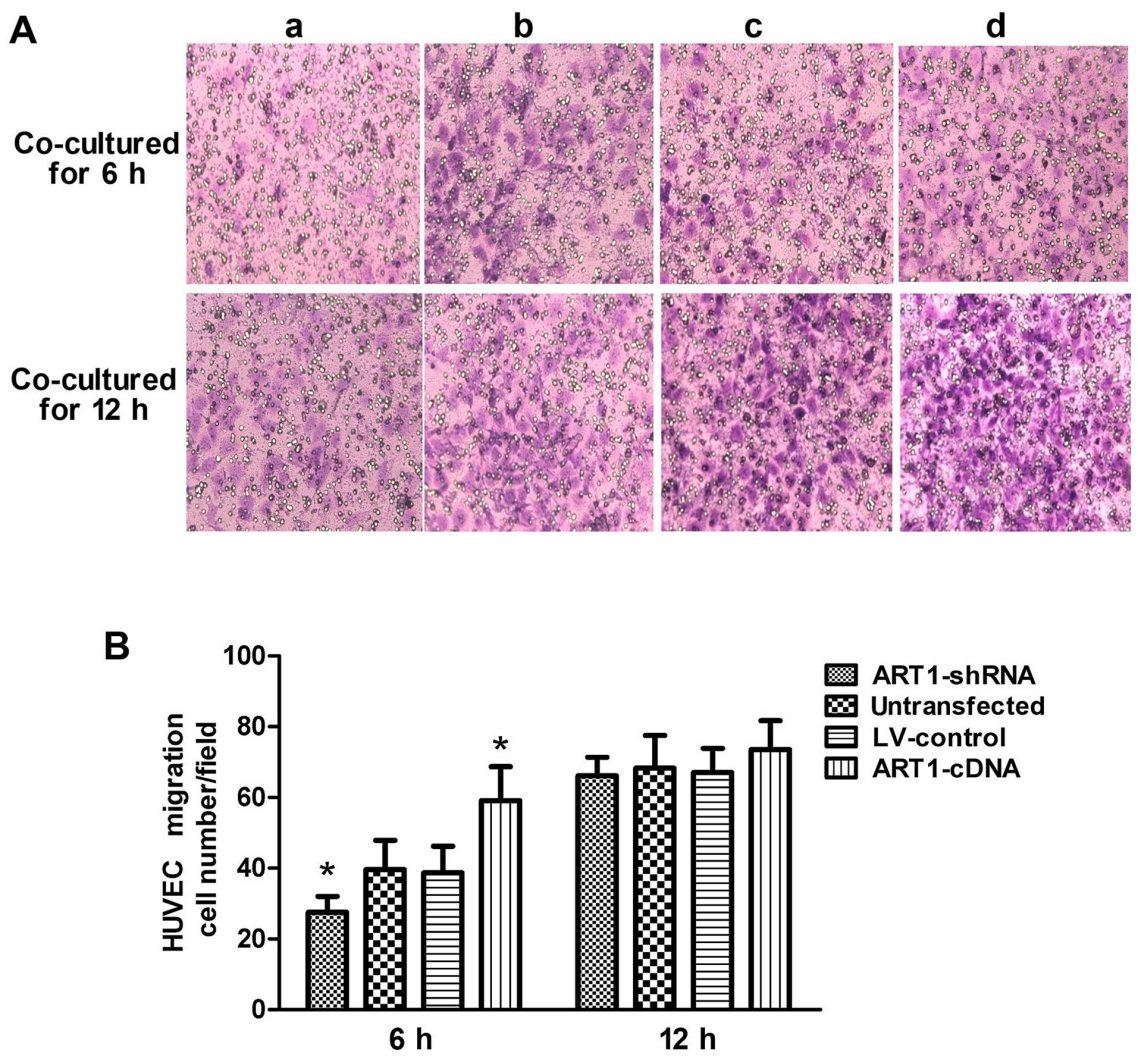
The effect of argenine-specific adenosine diphosphate (ADP)-ribosyltransferase 1 (ART1)-mediated LoVo cells on HUVEC migration. (A) The migratory ability of human umbilical vein endothelial cells (HUVECs) co-cultured with ART1-mediated LoVo cells. (a) HUVECs co-cultured with ART1-shRNA LoVo cells; (b) HUVECs co-cultured with the untransfected LoVo cells; (c) HUVECs co-cultured with LV-control LoVo cells; (d) HUVECs co-cultured with the ART1-cDNA LoVo cells. (B) The comparison of migration of HUVECs co-cultured with ART1-mediated LoVo cells afer 6 and 12 h. ^*^P<0.05 vs. untransfected LoVo cells or lentivirus (LV)-control LoVo cells; n=3 experiments.

**Figure 6:**
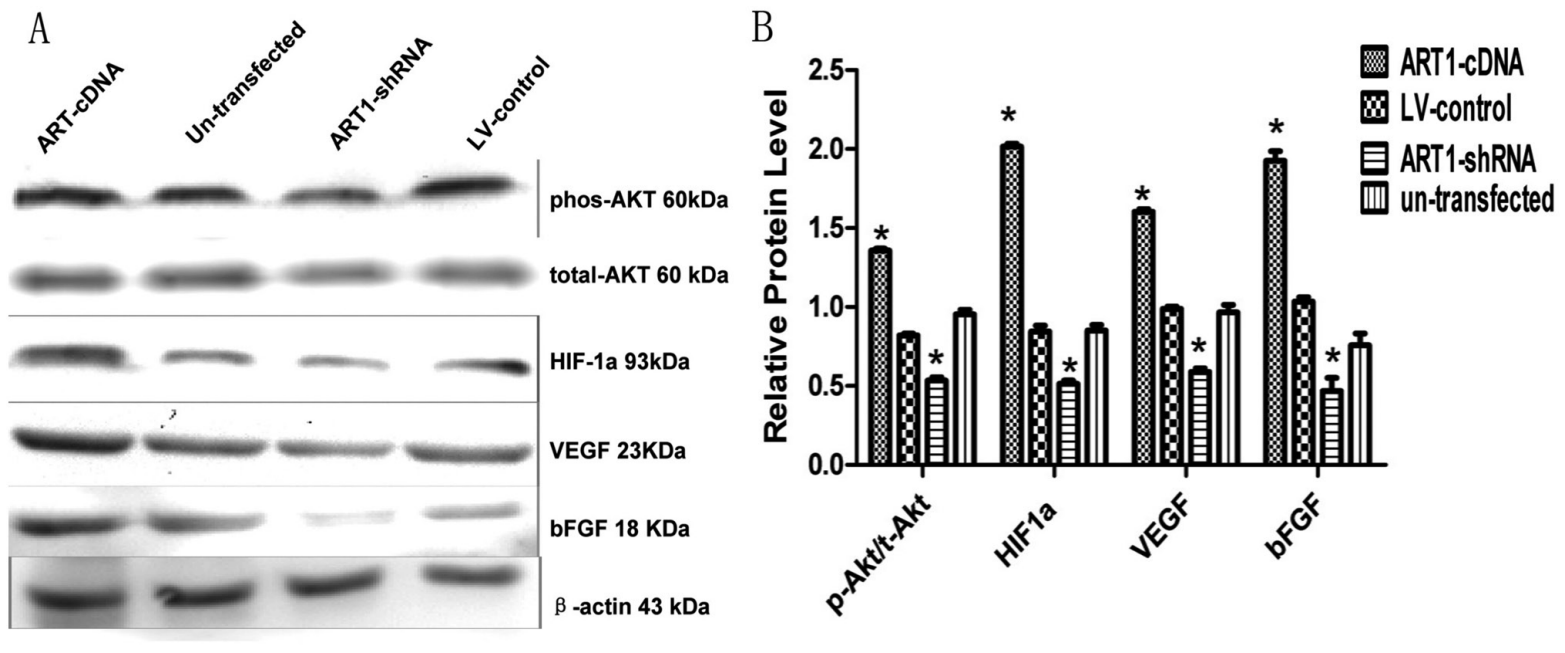
Arginine-specific adenosine diphosphate (ADP)-ribosyltransferase 1 (ART1) affects total-Akt, p-Akt, HIF-1α, VEGF and bFGF expression in LoVo cells. (A) The expression of total-Akt, p-Akt, hypoxia-inducible factor-1α (HIF-1α), vascular endothelial growth factor (VEGF) and basic fibroblast growth factor (bFGF) in ART1-mediated LoVo cells. (B) Comparison of Akt, p-Akt, HIF-1α, VEGF and bFGF expression in ART1-cDNA-transfected LoVo cells with that of ART1-shRNA-transfected LoVo cells, LV-control-transfected LoVo cells and untransfected LoVo cells. ^*^P<0.05 vs. untransfected LoVo cells; n=3 experiments.

